# Enhanced antimicrobial efficacy of hydroxyapatite-based composites for healthcare applications

**DOI:** 10.1038/s41598-024-76088-4

**Published:** 2024-11-02

**Authors:** Maher Hassanain, Hamdy Maamoun Abdel-Ghafar, Hamed I. Hamouda, Fouad I. El-Hosiny, Emad M. M. Ewais

**Affiliations:** 1 Central Metallurgical Research and Development Institute (CMRDI), PO Box 87, Helwan, Cairo 11421 Egypt; 2 Chemistry Department, Faculty of Science, Ain Shams University, Abbassia, Cairo, 11566 Egypt; 3 Dalian Engineering Research Center for Carbohydrate Agricultural Preparations, Liaoning Provincial Key Laboratory of Carbohydrates, Dalian Institute of Chemical Physics, Chinese Academy of Sciences, CAS, Dalian, 116023 China; 4 Processes Development Department, Egyptian Petroleum Research Institute, Nasr City, Cairo 11727 Egypt

**Keywords:** Hydroxyapatite, Wet chemical precipitation, Copper, Zinc, Silver, Anti-microbial, Microbiology, Health care, Nanoscience and technology

## Abstract

Hydroxyapatite (HAp) and hydroxyapatite-based materials show promising potential in the healthcare sector due to their distinctive properties such as biocompatibility, antimicrobial efficacy, non-toxicity, and robust mechanical characteristics. This makes HAp materials play an important role in hindering infection spreading in healthcare provider institutions. This study assesses the antimicrobial efficacy of the developed hydroxyapatite-based composites incorporating copper, zinc, and silver nanoparticles. The synthesized HAp and its modified composite variants (Cu/HAp, Zn/HAp, and Ag/HAp) with varying ratios ranging from 0 to 15% (wt) were characterized using XRD, XPS, SEM, and TEM analyses. Furthermore, the antibacterial and antifungal properties of the synthesized HAp and HAp-based composites were evaluated. The antibacterial effectiveness of the HAp and its composites was evaluated using a modified disc diffusion test, where the resulting inhibition zones on the agar surface were observed. All the HAp and HAp-based composites (HAp, Cu/HAp, Zn/HAp, and Ag/HAp materials) elicited in the formation of inhibitory zones. The most substantial inhibition values were observed for the 5% Ag/HAp formulation, with values of 19.7 and 13.8, against E. coli and S. aureus, respectively. The 5% Ag/HAp concentration may strike an ideal balance, providing high antimicrobial activity without adverse effects on biocompatibility or material stability. These findings underscore the recommendation of the proposed HAp-based composites for infection control measures through their application on medical instruments, textiles, healthcare personnel attire, and patient garments.

**Supplementary Information:**

The online version contains supplementary material available at 10.1038/s41598-024-76088-4.

## Introduction

Preventing cross-infection is a critical concern in healthcare organizations. While stringent barriers and conventional procedures are crucial and frequently used, they are insufficient to entirely eliminate the risk of infection. Reducing the presence of pathogenic organisms in the patient environment is essential, as they can be found on surfaces such as handrails, doorknobs, faucets, beds, tables, linens, curtains, and clothing. This reduction is vital for preventing hospital-acquired and healthcare-associated infections.^1–6^.

Hospital-related infections are often caused by pathogens present in the hospital environment, including on curtains and blankets. Because many hospital patients have suppressed immune systems, appropriate infection control procedures are essential. Numerous infectious microbes, including bacteria, viruses, and fungi, contribute to hospital-related diseases. Given that germs can easily adhere to medical equipment and subsequently spread to patients, the risk of cross-infection is high.

Despite its numerous applications, including in fertilizer industries, catalysis, protein chromatography, pharmaceuticals, water treatment, and bone and tooth repair, pure hydroxyapatite (HAp) is extremely limited due to its fragility. Significant efforts have been made to modify HAp with polymers, as natural bone is a composite material primarily composed of needle-shaped HAp nanoparticles, which account for about 65% of bone weight^7^, and collagen fibers^8,9^. Ultrafine HAp powder is commonly used to produce high-quality HAp bioceramics for artificial bone replacement^10,11^. HAp is stoichiometric, biocompatible, osteoconductive, non-toxic, non-inflammatory, non-immunogenic, and biologically active^12^. These properties allow it to chemically bind directly to living tissue^12^. Micro-HAp is available in various forms, including thin film coatings, powder, porous scaffolds, and dense blocks^13^. However, its low antimicrobial efficacy compared to hydroxyapatite-based composites limits its broader application in different fields.

Despite the unique properties and unexpected results from the direct application of metal nanoparticles in various fields such as medicine, agriculture, and energy, a global threat has emerged due to the potential uncontrolled release and inadequate monitoring of these nanoparticles, posing risks to human health and the environment^14,15^. Immobilizing metal nanoparticles in safe and easily recognizable materials, such as biocompatible composites^16,17^, substrates, membranes^18^, and polymers^19^, makes them more suitable for a wide range of industrial applications.

HAp plays a crucial role in the healthcare sector due to its simple synthesis process, biocompatibility, bioactivity, and ability to be loaded with various nanoparticles^20^. The antimicrobial properties of metal-doped HAp composites, such as those incorporating divalent (Ni and Zn) and trivalent (Al and Fe) metals by wet-impregnation technique, are significantly enhanced and surpass those of pure HAp^21^. Phatai et al.^22^ found that the synthesized mesoporous Zn^2+^/Ag^+^ doped HAp nanoparticles through ultrasonic coupled sol–gel techniques showed high antibacterial activity against E. coli, P. aeruginosa, S. aureus, B. cereus and B. subtilis. The synthesized nano-HAp doped with either Ag^+^ or Zn^2+^ by sol–gel precipitation method showed a promising antibacterial activity against an important microorganism related to oral infections, Aggregatibacter actinomycetemcomitans^23^. The derived HAp from chicken bone dopped with Ag nanoparticles demonstrated excellent antibacterial activity against Klebsiella pneumoniae, Staphylococcus aureus, Bacillus cereus with inhibition zones of 28 mm, 26 mm, and 24 mm, respectively^24^.

This work introduce a comparative study of HAp doped with three different metals nanoparticles with demonstrating the antimicrobial activity for both bacteria and fungi. This involves the preparation and characterization of HAp and HAp decorated with different metals, including Cu, Zn, and Ag. The pure HAp and metal-doped HAp were characterized using XRD, XPS, SEM, and TEM, and their antibacterial and biological properties were evaluated.

## Experimental

### Materials

All used analytical grade chemicals were used as received without extra modification. Ammonium Dihydrogen Orthophosphate (NH_4_H_2_PO_4_, assay ≥ 99.0%, was supplied from Alpha Chemika – India, Calcium Hydroxide from Qualikems-India, Ca(OH)_2_ from Qualikems-India, absolute ethanol was purchased from Sigma-Adrich- Germany. Ammonia solution was purchased from Honeywell-Seelze GmbH –Germany, while copper nitrate (Cu(NO_3_)_2_, zinc nitrate (Zn(NO_3_)_2_), and silver nitrate (AgNO_3_) with min assay ≥ 99.8% was purchased from Nice Chemicals. Aqueous solution was prepared using bi-distilled water during the whole period of the experiment.

Nutrient broth (N.B.) medium and Luria–Bertani (L.B.) medium were ordered from Fluka, BioChemika, Spain; Merck, Germany; and OXOID, U.K., respectively. Tetracycline antibiotic was purchased from Sigma-Aldrich, St. Louis, MO, USA. Dimethyl sulfoxide (DMSO), Nystatin antifungal, and phosphate-buffered saline (PBS) were obtained from Solarbio Science & Technology, Beijing, China. The Potato Dextrose Agar (PDA) medium consisted of 200 g of potato extract, 20 g of agar, and 15 g of glucose per 1000 ml of distilled water, with the pH adjusted to 7.0. The Yeast Extract Sucrose (YES) medium was prepared by combining 2 g of yeast extract, 15 g of sucrose, and 2 g of agar per 100 ml of distilled water for the fungal disc diffusion test.

### Methods

#### Preparation of hydroxyapatite (HAp)

Amorphous HAp was synthesized using a wet chemical precipitation method. Briefly, Ca(OH)₂ (0.1 M) and NH₄H₂PO₄ (0.06 M) were separately dissolved in bi-distilled water, with the pH of both solutions maintained at 11.0 using ammonia solution. The NH₄H₂PO₄ solution was added dropwise to the Ca(OH)₂ solution over 2 to 3 h at a temperature above 90 °C to form a white suspension with a gelatinous precipitate. The solution was allowed to cool to room temperature with continuous stirring for 24 h, then filtered. The precipitate was washed three times with bi-distilled water and ethanol, dried at 105 °C for 12 h, and then calcined at 600 °C for 2 h.

#### Preparation of HAp-based composites

The obtained HAp was decorated with Cu, Zn, and Ag nanoparticles via the wet-impregnation technique assisted by ultrasonication (SONICA 2400MH S3, 50/60 Hz, Italy, 305 Watt). Briefly, a homogeneous suspension of HAp was prepared by dispersing 1.0 g of HAp in 100 mL of ethanol and ultrasonicated for 30 min. During ultrasonication, the desired volume of 0.1 M Cu(NO₃)₂, Zn(NO₃)₂, or AgNO₃ solution was added dropwise to achieve the target metal ratios of 5%, 10%, and 15% by weight on the surface of HAp. The HAp/metal precursor mixture was then dried at 70 °C for 12 h to completely evaporate the ethanol, followed by calcination at 600 °C for 2 h.

### Characterization

The phase compositions of all synthesized HAb composite samples were determined by X-ray diffraction (XRD, X Pert pro Panalytical company, 45kV 40 mA, Source Cu 1.54A). The phase identification was confirmed by comparing the diffraction pattern of all samples with standard reference of (JCPDS cards). A scanning electron microscope (SEM) equipped with energy dispersive spectroscopy (EDS) was used to examine the morphology of HAp and the decorated HAp with metals using Field Emission Scanning Electron Microscope (Sigma 300VP) model Sigma 300VP company Zeiss. A transmission electron microscope (TEM, modelL Jeol Jem 2100 company Jeol) was utilized to assess the nanoparticles’ morphology. X-ray photoelectron spectroscopy (XPS) was conducted to establish the elemental composition and the chemical states of the samples. The XPS wide and narrow scan spectra were acquired using an X-ray photoelectron spectrometer. XPS was collected on K-ALPHA (Thermo Fisher Scientific, USA) with monochromatic X-ray Al K-alpha radiation -10 to 1350 e.v spot size 400 micro m at pressure 10–9 mbar with full spectrum pass energy 200 e.v and at narrow spectrum 50 e.v. The particle size analysis was carried out using a laser particle size analyzer, model COR-2000 designed and manufactured by “CORDOUAN”.

### In vitro anti-microbial activity study

#### Test microorganisms and media

The inhibitory action of the synthesized HAp-based composites was tested against standard microbial strains, including five species of pathogenic bacteria. These comprised two Gram-positive bacteria: Staphylococcus aureus ATCC 13,565 and Bacillus cereus EMCC 1080, and three Gram-negative bacteria: Escherichia coli O157.

ATCC 51,659, Pseudomonas aeruginosa NRRL B-272, and Salmonella typhi ATCC 25,566. All bacterial strains were purchased from the Holding Company for Biological Products and Vaccines (VACSERA), Egypt. The stock cultures were grown on nutrient agar slants for 24 h at 37℃ and then stored at – 80 ℃ until use.

For antifungal determination, three fungal species were utilized: Penicillium verrucosum BFE 500, Aspergillus parasiticus SSWT 2999, and Fusarium verticelloides ITEM 10,027. These fungal strains were obtained from the Applied Mycology Department at Cranfield University, U.K. After being cultured on potato dextrose agar slants for five days at 25℃, the stock cultures were refrigerated until needed. Untreated microbial solutions, without HAp nanocomposites, were used as negative controls.

#### Evaluation of anti-bacterial potency

An agar disc diffusion assay was conducted using the Kirby-Bauer technique to evaluate the antimicrobial activity of various concentrations and compositions of synthesized HAp-based composites^25^. This method is widely used for a qualitatively assessing the antimicrobial susceptibility of bacteria^26^. The disc diffusion method is a practical and widely used tool for initial antibiotic susceptibility testing and assessing the synergistic effects of multiple antimicrobial agents. However, unlike broth dilution methods, it does not provide a precise quantitative measure of the antimicrobial concentration required to inhibit bacterial or fungal growth^27,28^.

The composites tested included Cu/HAp, Zn/HAp, and Ag/HAp at 0%, 5%, 10%, and 15% loading ratios of Cu, Zn, and Ag. The aim was to determine the concentrations of composites capable of exhibiting antimicrobial activity against the five bacterial strains. Prior to the antibacterial assay, the samples were sterilized at 110 °C.

Sterilized discs (6 mm) made from Whatman No. 1 filter paper were loaded with the synthesized HAp-based composites and dried completely under sterile conditions. According to a previous study^29^, 2.5 g of L.B. powder medium was added to 100 mL of distilled water, the pH was adjusted to 7.4, and the solution was sterilized by autoclaving at 121 °C for 20 min.

The agar surfaces of three petri dishes were inoculated with each concentration by spreading a 100 µL suspension of the five pathogenic strains across the entire agar surface using a sterilized cotton swab. After inoculation, the prepared discs containing various concentrations of the nanocomposites were placed separately onto the seeded agar surface under aseptic conditions using sterile forceps. Discs loaded with sterile DMSO served as negative controls, while standard tetracycline solutions (500 µg/mL) were used as positive controls.

The inoculated plates were then refrigerated at 4 °C for 3 h to enhance the diffusion of the antimicrobial agents, followed by incubation at 37 °C for 24 h to observe the inhibition zones. The antimicrobial efficacy of the prepared M/HAp composites was determined by measuring the diameter of the inhibition zones (clear zones, mm), including the diameter of the paper discs. Positive antimicrobial activity was indicated by the presence of clear zones, which resulted from the inhibition of bacterial growth by the M/HAp composite materials. The specimens tested included HAp, 5% Cu/HAp, 10% Cu/HAp, 15% Cu/HAp, 5% Zn/HAp, 10% Zn/HAp, 15% Zn/HAp, 5% Ag/HAp, 10% Ag/HAp, 15% Ag/HAp, as well as negative and positive controls.

#### Evaluation of antifungal potency

The antifungal activity of synthesized HAp-based composites with varying concentrations and compositions was evaluated against the tested fungal strain using the disc diffusion assay. A 50 µL aliquot of each fungal suspension (about 2 × 10^8^ CFU/mL) was added to petri plates containing YES medium, and the mixture was evenly spread using a sterile L-glass rod. As in the antibacterial test, sterilized filter paper discs (6 mm) were loaded with the synthesized HAp-based composites and allowed to dry thoroughly in a sterile environment.

The discs, containing various concentrations of the HAp-based composites, were placed onto the fungal cultures on YES plates using sterile forceps. The setup included a negative control (only DMSO) and HAp-based composites at different concentrations (0–15%). Nystatin (1000 units/mL), a commonly used fungicide, served as the positive control. The inoculated plates were incubated at 25°C for 48–72 h. After the incubation period, the degree of inhibition (mm) against the tested fungus was measured to assess antifungal activity^30^.

## Results and discussion

### XRD pattern

**Fig. 1:**
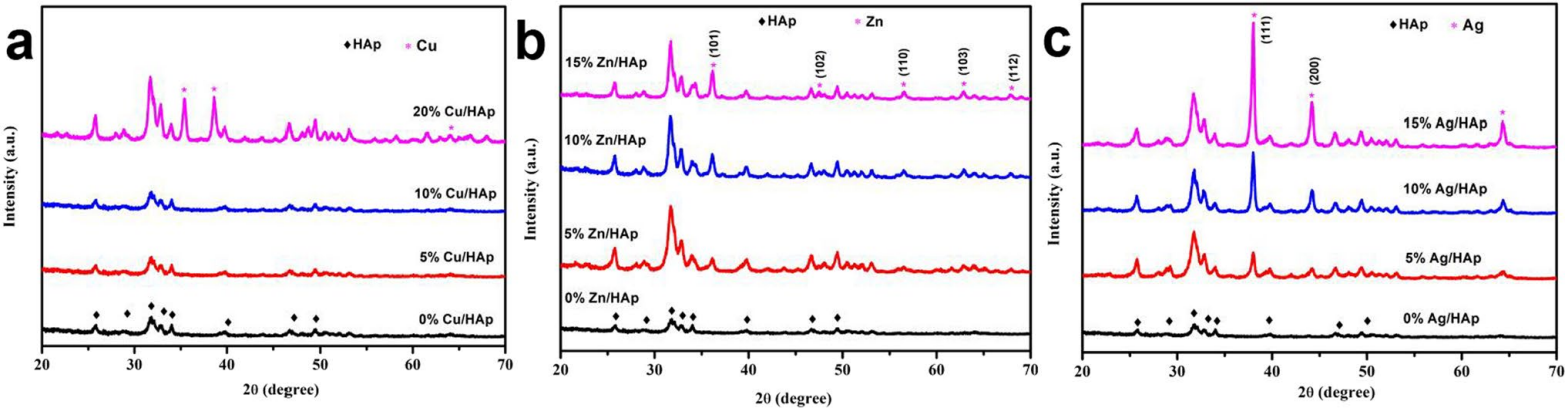
Characteristics of the XRD pattern of the synthesized HAp-based composites: (**a**) Cu/HAp, (**b**) Zn/HAp, and, (**c**) Ag/HAp composites, at 0, 5, 10, and 15% loading ratio of metal nanoparticles.

Figure 1 shows the XRD analysis of the HAp-based composites. The XRD spectrum of the HAp base sample (the baseline) shows broad diffraction peaks at 25.9°, 28.25°, 31.9°, 33°, 34.2°, 39.96°, 46.85°, and 49.5°. All reflections are characteristics of the hexagonal phase of pure HAp [Ca_10_(PO_4_)_6_(OH)_2_], according to the standard data (JCPDS No. 74-0566). The obtained XRD patterns are align with findings reported by Kim^31^, and Heidari^32^,^36^].

The XRD phase identification of the Cu/HAp composites was confirmed by comparing the diffraction pattern of all samples with the reference standard HAp (JCPDS 01-073-0294) and Cu-metal (JCPDS 00-045-0937) available in the system software. As shown in Fig. 1a, there are no significant phases changes in the XRD diffractograms of Cu-doped HAp as the ratio increases from 5 to 15% (wt), indicating that the crystal structure of Cu-doped HAp remains stable during sintering when the copper concentration is less than 15%. Compared to the pure HAp samples, the position of the XRD peaks of the Cu-doped sintered HAp samples changed, e.g., the (300) and (211) peaks moved towards the small angle. Since the radius of copper ions is smaller than that of calcium ions, a contraction of unit cell parameters is expected when Cu replaces Ca sites in HAp, causing the XRD peaks to shift to larger angles^33,34^. However, in this study, an increase in the doped Cu amount resulted in the XRD peaks shifting to smaller angles, as shown in Fig. 1a. This indicates that the copper ions in the apatite target the OH positions and not the Ca positions^35^.

The XRD analysis for the Zn/HAp composites was presented in Fig. 1b. The XRD spectra indicates broad diffraction peaks at 25.9°, 28.25°, 31.9°, 33°, 34.2°, 39.96°, 46.85°, and 49.5°. All reflections are characteristic of the hexagonal phase of HAp [Ca_10_(PO_4_)_6_(OH)_2_] according to standard data (JCPDS No. 74–0566) and others^31,36^. The diffraction pattern shows characteristic peaks of ZnO nanoparticles peaks at 36.2°, 47.51°, 56.6°, 62.86°, and 67.89° corresponding to (101), (102), (110), (103), and (112) reflections, according to ZnO wurtzite structure (PDF code no: 00–036-1451). Similar diffraction peaks were stated by Koller and others^37,38^. Compared to the reference chart of zinc oxide, the growth peak increases with increasing zinc concentration from 0 to 15%.

The XRD patterns of the Ag/HAp composites formed at different concentrations of AgNO_3_ with loading ratios 0, 5, 10, and 15% with respect to the weight of HAp, as shown in Fig. 1c. To identify and compare the obtained peaks of the Ag/HAp XRD pattern, the standard HAp (JCPDS 09–0432) and metallic Ag (JCPDS 04–0783) XRD spectra were used. The diffraction pattern shows peaks at the values 2θ 25.9°, 29.0°, 31,8°, 32,2°, 33,0°, 34,1°, 39.9°, 46,7°, 48,1°, 49,5°, 50,5°, 51.3°, and 53.2°, which are associated with (002), (210), (211), (112), (300), (202), (310), (222), (312), (213), (321), (410), and (004) floors of the HAp network^39^. Additional peaks at ~ 38.2° and 44.4° correspond to the (111) and (200) lattice planes of face-centered cubic (fcc) of Ag nanoparticles. Rajendran et al. reported the formation of HAp and Ag/HAp nanocomposites with XRD pattern that agree with these findings^40^. Furthermore, no peaks other than HAp and Ag are observed in all Ag/HAp nanocomposites, indicating that the chemical reduction and calcination processes did not introduce any impurities with pure phases. It is also be seen that the intensity of specific Ag peaks in the Ag/HAp nanocomposite samples gradually increases with the concentration of AgNO_3_.

### Particle size distribution analysis

**Fig. 2:**
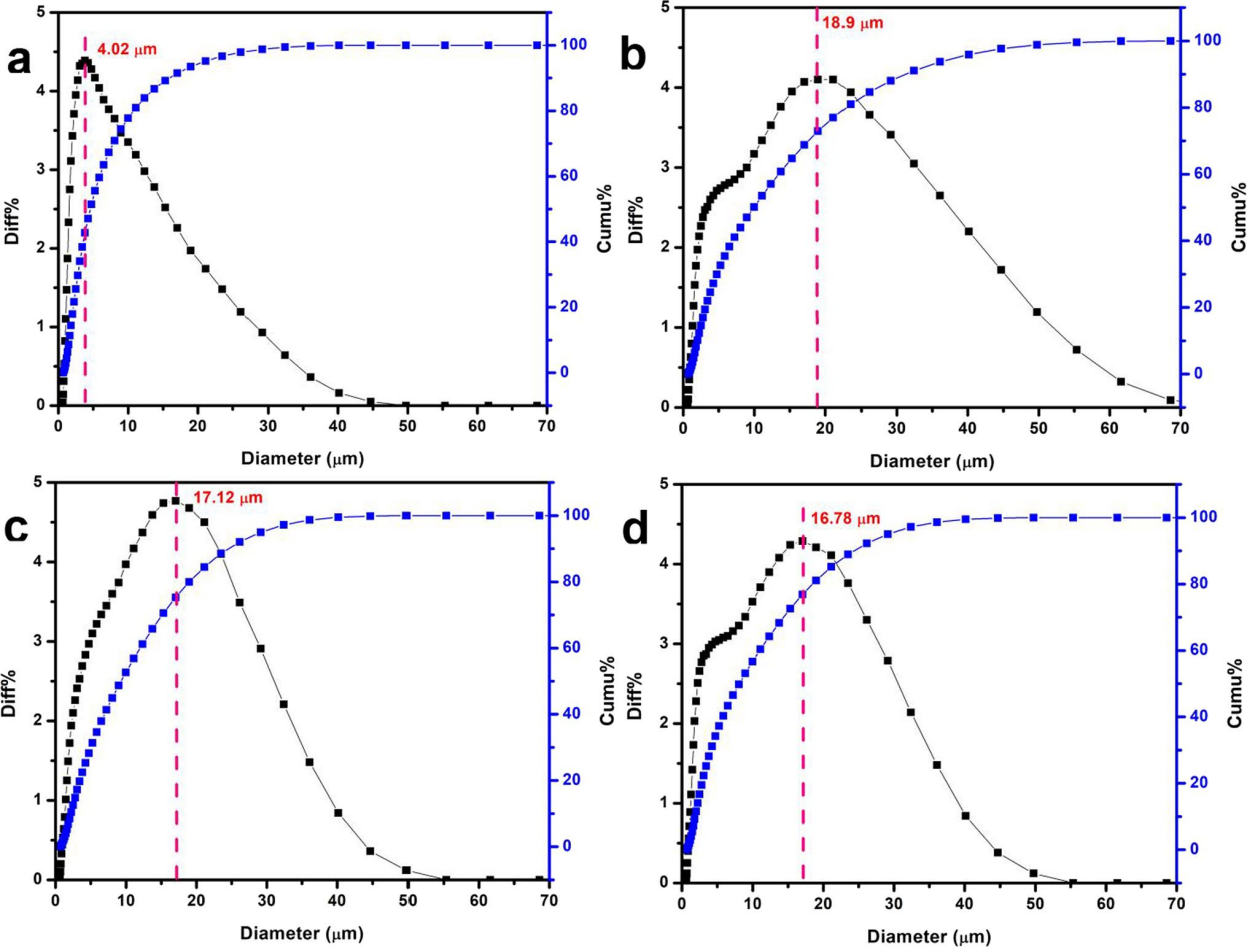
Volume fraction differences (%) and cumulative fractions (%) of the synthesized pristine HAp (**a**), and 10% (**b**, **c**, and **d**) of Cu/HAp, Zn/HAp, and Ag/HAp, respectively.

The particle size distribution (PSD) analysis of the synthesized HAp and metal-based HAp composites is illustrated in terms of cumulative and volume fraction differences as shown in Fig. 2, and Table S1 to S3 in the Supplementary Information (SI). Figure 2a-d compares the PSD of the pristine HAp with that of HAp composites decorated with 10 wt% of Cu, Zn, and Ag. The PSD data show a nonsymmetric distribution of the obtained pure HAp and other composites, with noticed increment in the modal diameter (the most common size range) from 4.02 µm of the pure HAp to 18.9 µm, 17.12 µm, and 16.76 µm of the 10 wt% Cu/HAp, Zn/HAp, and Ag/HAp, respectively. This increase in particle size may be attributed to the agglomeration of particles in the presence of metal nanoparticles, which occurs at a relatively low sintering temperature (600°C)^41,42^. In general, the decoration of HAp with metal NPs leads to agglomeration of crystallites at low sintering temperatures^43^.

### Surface morphology analysis

**Fig. 3:**
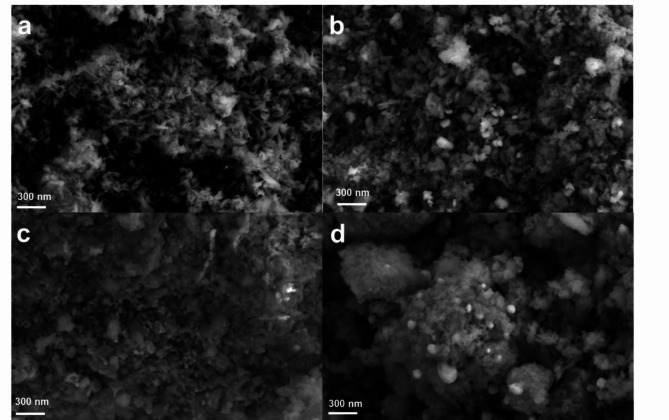
SEM analysis of the (**a**) pristine HAp, (**b**) 10% Cu/HAp, (**c**) 10% Zn/HAp, and (**d**) 10% Ag/HAp composites.

The surface morphologies of the pristine HAp and 10 wt% of the hydroxyapatite-based composites were investigated by SEM, as shown in Fig. 3 and Fig. S1. The SEM images show that the synthesized composites mainly consist of fine-grained particles with a homogeneous and uniform distribution of the components. The EDX analysis and SEM mapping of the elemental distribution are provided in the SI file (Fig. S2 and S3).

The SEM of the synthesized pristine HAp shows spherical particles allow for a high degree of packing (Fig. 3a), with most particles being submicron in size, as shown in Fig. S1 (a). The SEM morphology of the 10% Cu/HAp composite demonstrates a heterogeneous microstructure, which includes agglomerated particles of various shapes, as shown in Fig. 3b. The SEM analysis of the 10% Zn/HAp composites reveals that the precipitates have typical particle shapes without sharp angles, with the particles tending to agglomerate in spherical shapes (Fig. 3c). SEM images of the prepared 10% Ag/HAp composite, shown in Fig. 3d, and with different magnifications in Fig. S1 (d), reveal an irregular grain-like morphology with an interconnected porous network. Additionally, spherical AgNPs are clearly observed decorating the surface of the HAp particles.

**Fig. 4:**
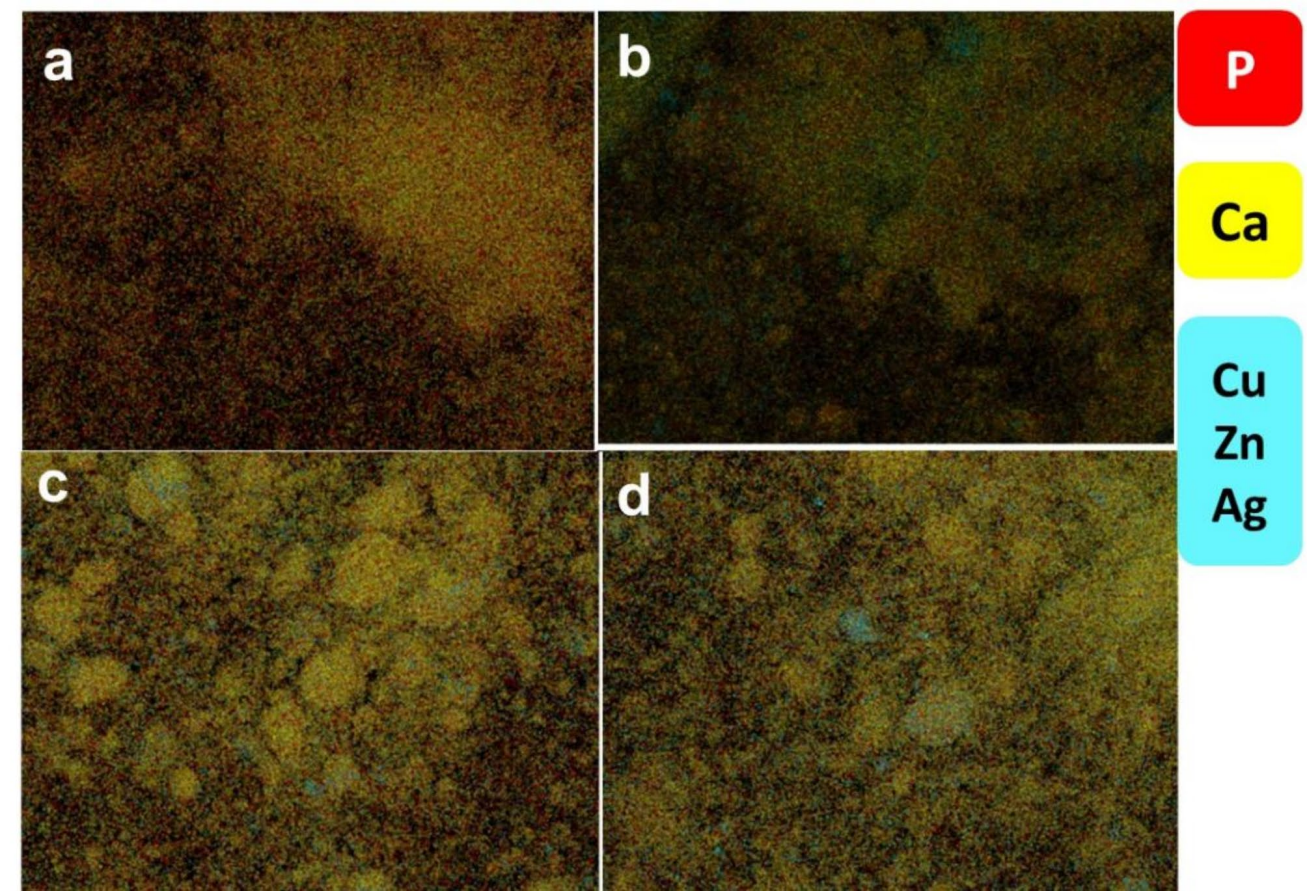
SEM–EDX mapping of the elemental distribution of the (**a**) pristine HAp, (**b**) 10% Cu/HAp, (**c**) 10% Zn/HAp, and (**d**) 10% Ag/HAp composites.

The elemental analysis of the pristine HAp (Fig. S2a) shows that the calcium to phosphorus (Ca/P) atomic ratio is 1.69 which is close to the stochiometric value in pure hydroxyapatite (Ca/P = 1.67). This indicates that the synthesized pristine HAp is of high purity. This ratio shifts with the doping of Cu, Zn, and Ag, likely due to side substitutions or reactions during the wet impregnation process. The obtained weight ratios of the doped metal nanoparticles are closely aligned with the intended doping ratios. The SEM–EDX mapping analysis (Fig. 4 and Fig. S3) of the HAp-based composites with 10% of Cu, Zn, and Ag shows a homogeneous distribution of the NPs on the surface of the HAp.

### TEM analysis

**Fig. 5:**
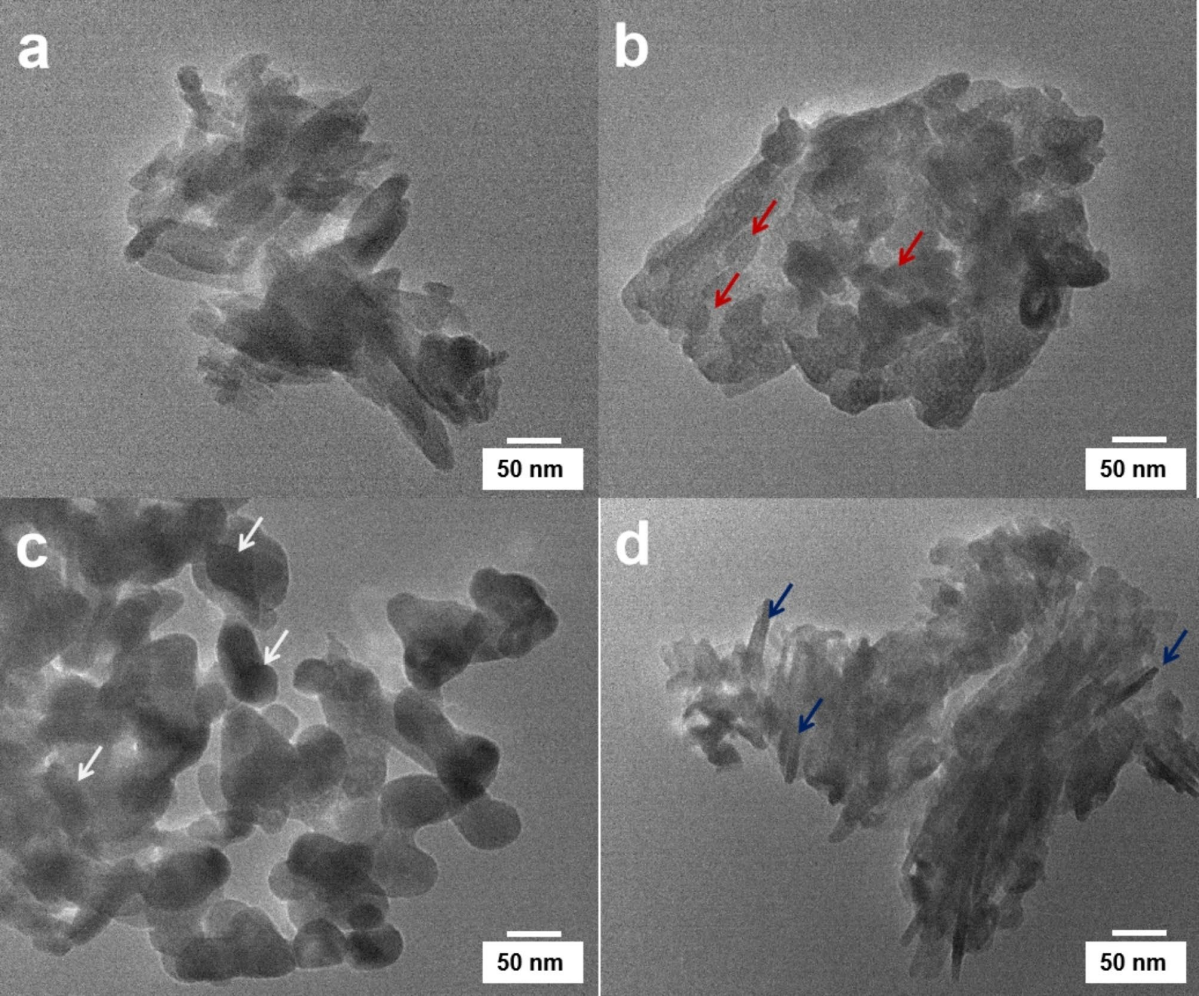
TEM analysis of the (**a**) pristine HAp, (**b**) 10% Cu/HAp, (**c**) 10% Zn/HAp, and (**d**) 10% Ag/HAp composites.

The TEM image of the crystallized HAp powder is shown in Fig. 5. The crystallized HAp powder exhibits a rectangular-like shape. These particles are agglomerated into nanospheres. The crystallized HAp powder exhibits, which further agglomerate into microspheres, as observed in the SEM images.

TEM images of the Cu/HAp composite show that the particles are more or less uniform in size and irregular in shape. These particles tend to agglomerate into larger clusters. In contrast, the 10% Zn/HAp composite exhibits a rod-shaped crystal structure (Fig. 5b). The dark-colored spots visible on the surface of the HAp crystals indicate the formation and decoration of AgNPs on the surface of the HAp particles (Fig. 5d). The 10% Ag/HAp crystals exhibit a needle-like structure.

### X-ray photoelectron spectroscopy

**Fig. 6:**
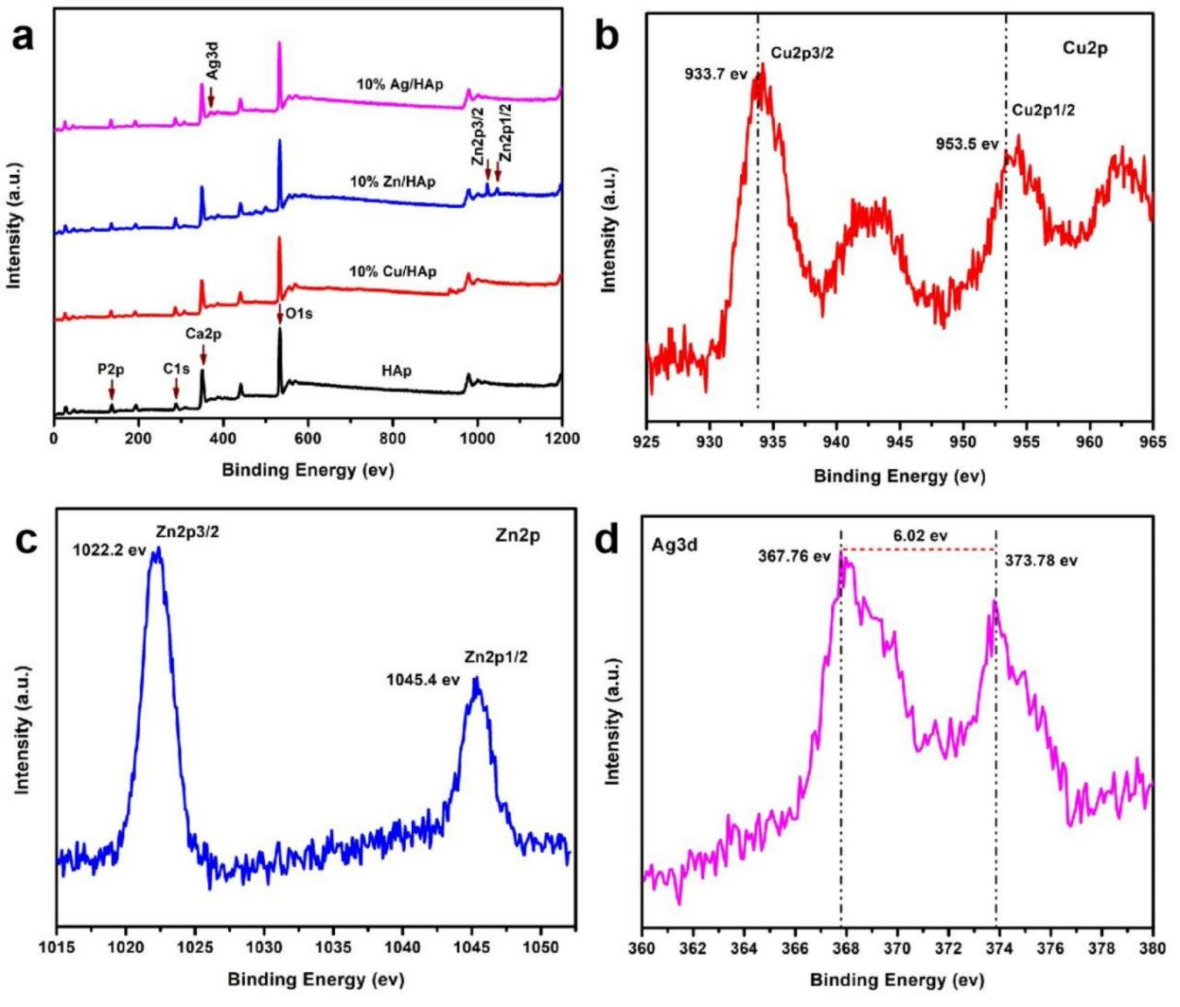
(**a**) XPS survey spectra, and high resolution XPS survey spectra of (**b**) 10% Cu/HAp, (**c**) 10% Zn/HAp, and (**d**) 10% Ag/HAp composites.

The chemical states and surface composition of the pristine HAp, 10% Cu/HAp, 10% Zn/HAp, and 10% Ag/HAp composites were analyzed using XPS, as shown in Fig. 6. A comprehensive survey revealed the presence of calcium (Ca2p), phosphorus (P2p), oxygen (O1s) and carbon (C1s), in addition to the characteristic peaks of Cu (2p3/2 and 2p1/2), Zn (2p3/2 and Ag (3d5/2 and 3d3/2) at 367.76 eV and 373.78 eV, respectively, as reported by others^44^.

The spin–orbit splitting of the dopped Cu, Zn, and Ag NPs on HAp, with two possible states having different binding energies, is shown in Fig. 6 (b, c and d). The spin–orbit splitting of Cu2p into Cu2p1/2 ad Cu2p3/2 with an energy difference of 19.8 eV is characteristic of Cu^2+^ ions^45^. Another copper peak 942.5 eV corresponds to the presence of Cu^2+^ on the surface of Cu/HAp composite^46^. The Zn2p peaks split into Zn2p3/2 and Zn2p1/2 with a spin–orbit coupling energy difference (ΔE) of 23.2 eV, as calculated form their XPS profile (Fig. 6c). The Ag3d peaks show a coupling energy difference of 6.02 eV between the Ag3d5/2 and Ag3d3/2 peaks. These ΔE values of Zn2p and Ag3d indicate that Zn^2+^ and Ag^+^ are the predominant species on their respective HAp-based composites, with zero valent states^17,47^.

### Anti-microbial activity study

#### Evaluation of Anti-bacterial potency

**Fig. 7:**
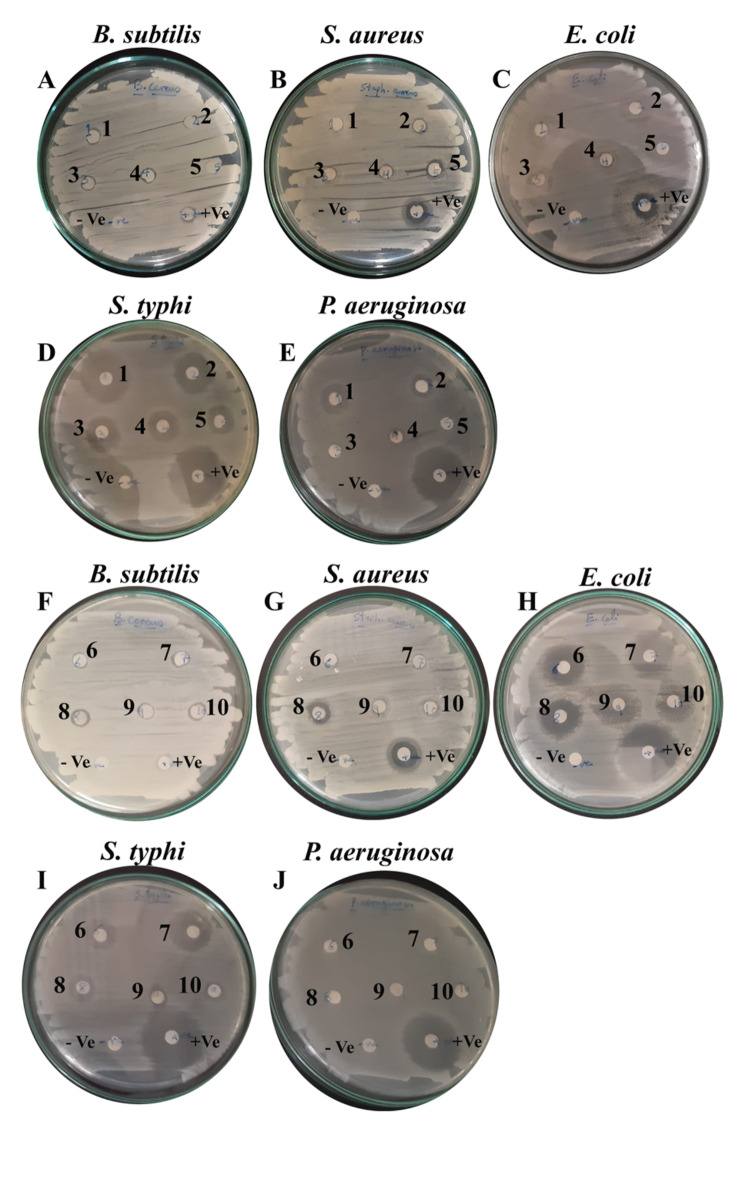
Agar disc diffusion method. The inhibition zone of the Cu/HAp, Zn/HAp, and Ag/HAp-based composites against different pathogenic bacteria: (**A** and **F**) *B*.* subtilis*, (**B** and **G**) *S*. *aureus*, (**C** and **H**) *E*. *coli*, (**D** and **I**) *S*. *typhi*, and (**E** and **J**) *P*. *aeruginosa*.

Nanomaterials have the potential to achieve bactericidal characteristics through many processes, such as change of the membrane, change of the cytoplasm and cell wall, or inhibition of the respiratory activity^48,49^. ZnO was already proven to have anti-microbial properties^18^. Besides HAp-based composites, additional zinc oxide in the synthesized nanocomposite demonstrated exceptional activity. It was expected that the prepared Zn/HAp- composites nanocomposite would display synergistic antibacterial properties. The primary antibacterial mechanisms of nano-ZnO particles include their ease of internalization into bacterial cells, the release of Zn^2^⁺ ions, the generation of reactive oxygen species (ROS), and disruption of the bacterial membrane^18^. The Agar diffusion assay estimates the anti-bacterial activity of the synthesized HAp-based composites. This method is popular because of its simplicity, cost-effectiveness, and ease of execution^26^. The antibacterial assay was carried out with bacterial culture of Gram-positive bacteria:* S*.* aureus*, *B*.* cereus,* and three Gram-negative bacteria *E*.* coli*, *S*.* typhi*, and *P*.* aeruginosa*, which is first spread on a Luria Bertani (L.B.) agar medium at 37 ℃ for 24 h. five pieces of 0.6 cm diameter of the developed dicks loaded with the synthesized composites were inserted in the plate, as shown in Fig. 7. The numbers on the disk indicate to the synthesized HAp-based composites; (1) pristine HAp, (2) 5% Cu/HAp, (3) 10% Cu/HAp, (4) 15% Cu/HAp, (5) 5% Zn/HAp, (6) 10% Zn/HAp, (7) 15% Zn/HAp, (8) 5% Ag/HAp, (9) 10% Ag/HAp, (10) 15% Ag/HAp composites. The antibacterial agent diffuses from the disc into the agar, there was a noticeable variance in the inhibitory zones of plates after 24 h of incubation between the control and the synthesized composites (Fig. 7) which is significantly greater than the discs of the pristine HAp alone and easily visible to the ordinary eye. The effectiveness of the synthesized composites is determined by measuring the zone of inhibition around the disc where bacterial growth has been prevented.

As shown graphically, no clear inhibition zone of *E. coli* and *S. aureus* wa*s* obtained for the unaltered HAp sample because the *S*.* aureus* and *E*.* coli* growth are still on and around the membrane. In contrast, a clear zone was observed with *P*.* aeruginosa* and* S*.* typhi* around the HAp sample. Interestingly, most of the prepared HAp-based composites, including unaltered HAp discs, exhibited high activity towards *E. coli* and *S. typhi*. In contrast, *B*.* cereus* and *S. aureus* showed the highest resistance among all tested materials, even compared to the positive control. The inhibition zone increased slightly from 5 to 10% composites, while there was no significant increase in the inhibition zone for the 15% prepared HAp-based composites.

**Fig. 8:**
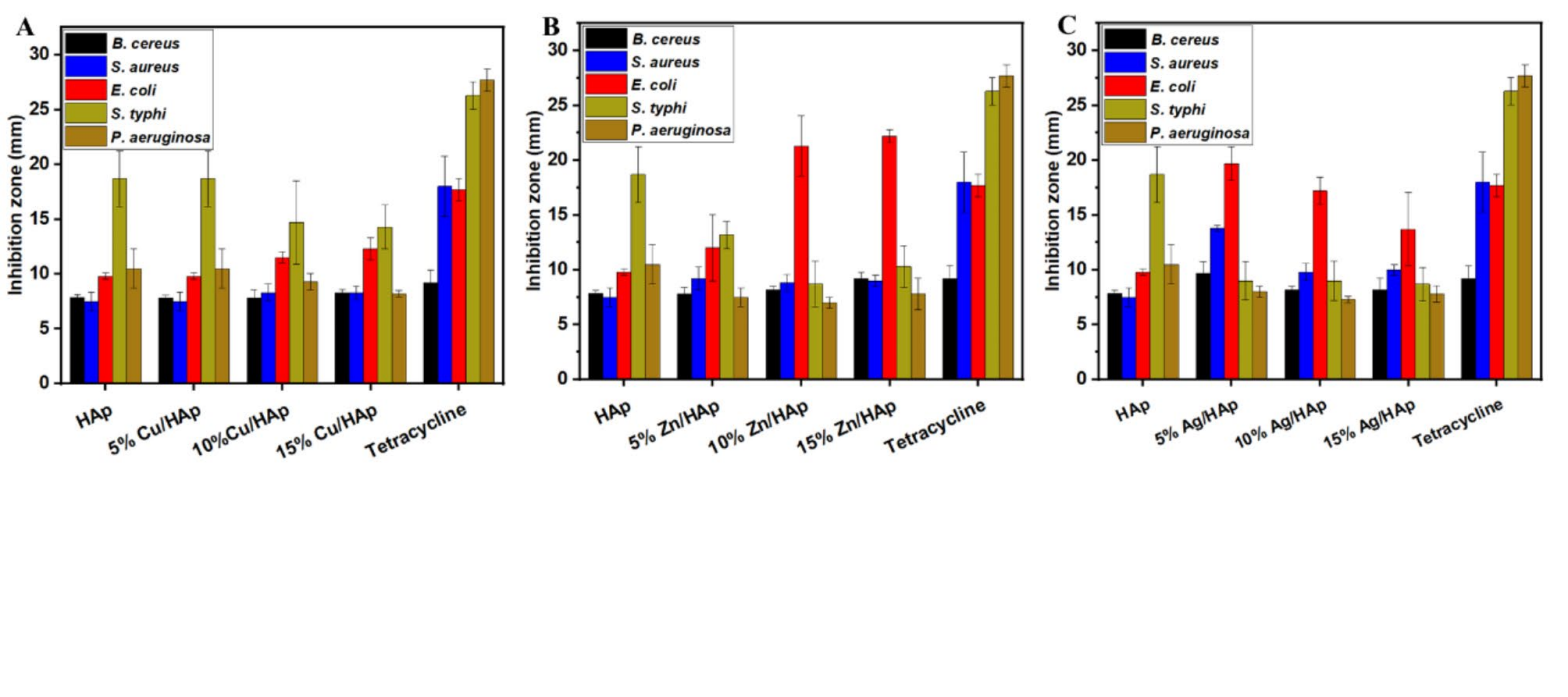
Anti-bacterial activity of HAp composites on pathogenic bacteria. (**a**) Cu/HAp, (**b**) Zn/HAp, and (**c**) Ag/HAp.

The inhibitory action for the Cu/HAp formulation was observed in the following order: *S*.* typhi*,* E*.* coli*,* P*.* aeruginosa*,* B*.* cereus*, and* S*.* aureus*. The highest inhibition values were recorded for the 5% Cu/HAp and 15% Cu/HAp formulations, with values of 18.7 mm and 12.3 mm, respectively, against *S. typhi* and *E. coli*, as shown in Fig. 7c, d. However, inhibition zones were barely observed for Cu/HAp against *B. cereus* and *S. aureus*. In the Zn/HAp formulation, inhibitory effects were noted in the following order: *E*.* coli*, *S*. *typhi*, *S*. *aureus*, *B*. *cereus*, and *P*. *aeruginosa*. The highest inhibition value was observed for the 15% Zn/HAp formulation, reaching 22.2 mm against *E*. *coli*, as shown in Fig. 7h and Fig. 8. Nevertheless, inhibition zones were hardly noticeably observed for Zn/Hap against *B*. *cereus* and *P*. *aeruginosa*. The inhibitory action for the Ag/HAp formula was observed in the following order: *E*.* coli*,* S*.* aureus*,* B*.* cereus*, *S*.* typhi*, and* P*.* aeruginosa*. The highest inhibition values were recorded for the 5% Ag/HAp formulation, with values of 19.7 mm and 13.8 mm, respectively, against *E. coli* and* S*.* aureus*. However, inhibition zones were barely observed for Cu/HAp against *B. cereus* and *S. aureus*. Notably, Ag/HAp exhibited a larger inhibition zone against *E*.* coli* compared to the positive control, as shown in Fig. 7h.

The Cu/HAp and Ag/HAp formulations generally exhibited similar inhibition patterns and produced the most significant inhibition zones against *E. coli*. Meanwhile, the Zn/HAp formulation demonstrated higher activity towards gram-positive (*S. typhi*) than gram-negative bacteria. Additionally, noticeable anti-bacterial properties and significant inhibition zones were observed around most of the composites prepared at lower concentrations (5%), as depicted in Fig. 7 and 8. These results suggest that silver (Ag) has a synergistic effect and can enhance anti-microbial activity more effectively than both Cu/HAp and Ag/HAp formulations.

### Evaluation of Antifungal potency

**Fig. 9:**
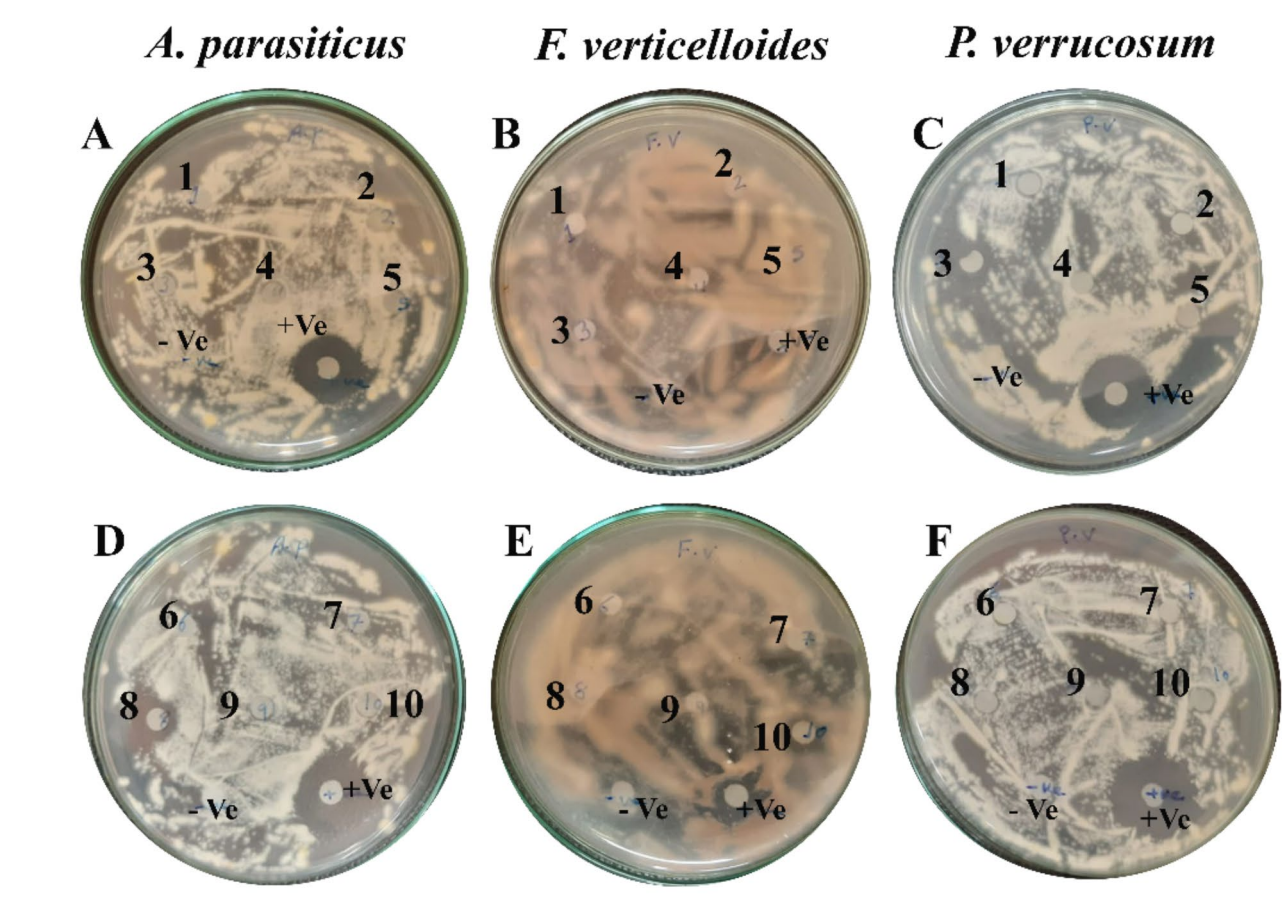
Agar disc diffusion method. The inhibition zone of the HAp-based composites against different pathogenic fungi (**A** and **D**) *A*. *parasiticus*, (**B** and **E**) *F*. *verticelloides*, and (**C** and **F**) *P*. *verrucosum*.

This strong adhesion might be owing to an electrostatic interaction between the ZnO/HAp Cu/HAp and Ag/HAp and bacterial cell membrane. The bacterial membrane consisted of peptidoglycan that had a negative charge. The preparation mechanism of ZnO/HAp Cu/HAp and Ag/HAp give them a positive charge. As a result of the electrostatic interaction, the nanocomposite could adhere to the microbial cells and cause additional disruption to the cellular walls of bacterial strains. This disruption in membrane integrity could be attributed to increased ROS generation in bacterial cells, which resulted in abnormalities and death. This experiment investigated the inhibitory effect of HAp composites on spore colony development of *A*. *parasiticus*, *F*. *verticelloides*, and *P*. *verrucosum* under in vitro conditions, as shown in Fig. 9 (A–F). The results indicated a suppression of spore development, leading to an inhibition zone by the HAp composite, demonstrating a significant antifungal effect on the tested fungi and their spore germination. Furthermore, increasing Cu, Zn, and Ag concentrations did not significantly enhance the antifungal efficacy against the pathogens. Therefore, we conclude that the 5% concentration represents the most cost-effective and environmentally friendly option and is preferable for use as an antifungal agent.

**Fig. 10:**
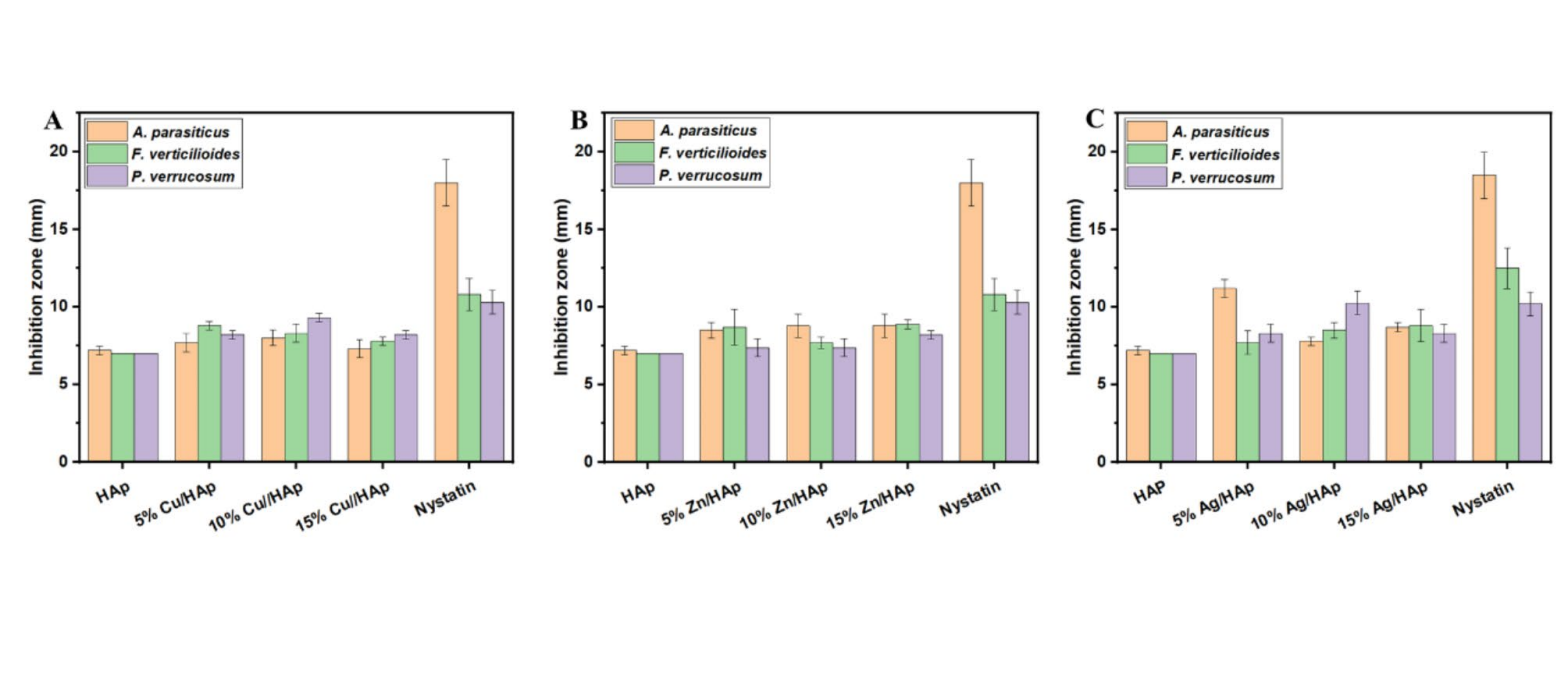
Images of the antifungal activity of the synthesized composites, (**a**) Cu/HAp, (**b**) Zn/HAp, and (**c**) Ag/HAp. on *A*. *parasiticus*, *F*. *verticelloides*, and *P*. *verrucosum*. The data represents the mean ± standard deviation of three different experiments.

The highest inhibition values were recorded for the 5% Ag/HAp and 10% Ag /HAp formulations, with values of 11.2 mm and 10.2 mm, respectively, against *A*. *parasiticus*, and *P*. *verrucosum*, as shown in Fig. 9 (A and C) and Fig. 10. No distinct inhibition zone was observed *A*. *parasiticus*, *F*. *verticelloides*, and *P*. *verrucosum* in the pure HAp samples, as fungal growth was evident on or around the membrane.

#### The anti-microbial mechanism

The antimicrobial mechanism of the Zn/HAp, Cu/HAp and Ag/HAp nanocomposite was evaluated using eight pathogens, focusing on bacteria and fungi common in human infections. The study demonstrated that the prepared nanocomposite effectively inhibited bacterial growth in *E. coli* and *S. typhi*, as evidenced by clear inhibition zone using agar well diffusion tests. Key points of the study include:

(1) **HAp Surface Features**: The wrinkled surface of HAp helps stabilize the nanocomposite in an aqueous medium, altering bacterial osmotic pressure and damaging cell membranes; (2) **Ag, Cu, ZnO Nanospheres**: These nanoparticles penetrate bacterial cell membranes, reducing cell viability through toxic interactions with the bacterial surface; (3) **Synergistic Effects**: The combined action of **Ag, Cu, or** ZnO and HAP disrupts cell walls through electrostatic interactions and oxidative stress, which impairs cellular functions and triggers bacterial cell death; (4) **ROS Generation**: The nanocomposite generates reactive oxygen species (ROS) that damage essential membrane enzymes and other cellular components, further contributing to bacterial cell death; (5) **Membrane Homeostasis Disruption**: Interaction with bacterial membranes affects cation transport and membrane stability, leading to cell death; (6) **Long-lasting Antibacterial Action**: The Zn/HAp, Cu/HAp and Ag/HAp nanocomposite remains stable in aqueous media, maintaining high dispersion and prolonged bactericidal activity due to the synergistic effects.

Overall, the Zn/HAp, Cu/HAp and Ag/HAp nanocomposite exhibits potent and sustained antibacterial properties, making it a promising candidate for biomedical applications.

There are different mechanisms by which we speculate that the antimicrobial agent inhibits the bacterial growth though these ways; For silver (Ag): silver ions (Ag^+^) disrupt microbial cell walls, bind to DNA, and interfere with cellular respiration and replication processes. Ag^+^ ions can also induce the generation of reactive oxygen species (ROS), which further damage microbial cells. The combination of these mechanisms makes silver particularly effective against a wide range of bacteria, fungi, and viruses.

Zinc (Zn): zinc ions (Zn^2+^) can inhibit the activity of essential microbial enzymes by binding to their active sites, interfering with nutrient uptake, and destabilizing cell membranes. Zinc’s ability to generate ROS also contributes to its antimicrobial action. Additionally, zinc can enhance the immune response, further helping to combat infections.

Copper (Cu): copper ions (Cu^2+^) can cause membrane damage, protein denaturation, and DNA disruption in microbial cells. Like silver and zinc, copper ions are also capable of generating ROS, leading to oxidative stress and cell death in bacteria. Copper’s broad-spectrum antimicrobial activity makes it effective against bacteria, fungi, and viruses.

The enhanced antimicrobial activity observed in the 5% Ag/HAp formulation compared to other metal-doped hydroxyapatite (HAp) composites, we speculate that might be due to different reasons as; (1) the 5% Ag/HAp formulation likely provides an optimal balance between silver content and the hydroxyapatite matrix, allowing for a sustained release of Ag + ions. The antimicrobial efficacy of silver is highly dependent on its ability to release ions, which interact with bacterial cell membranes, leading to structural disruption and cell death. A 5% doping level might represent a concentration where sufficient silver ions are available to exert a potent antimicrobial effect without compromising the structural integrity or biocompatibility of the HAp matrix; (2) additionally, the silver nanoparticles may be more uniformly distributed across the HAp surface, maximizing the contact area with microbial cells, which could enhance the interaction between the Ag/HAp composite and the microbial cells, leading to increased cell membrane disruption, (3) higher concentrations of silver may result in cytotoxic effects on human cells or reduce the mechanical properties of the composite. The 5% Ag/HAp concentration may strike an ideal balance, providing high antimicrobial activity without adverse effects on biocompatibility or material stability, (4) reducing silver, zinc or copper content in nanoparticle formulations lowers production costs while maintaining antimicrobial effectiveness, especially when combined with materials like HAp. This also lessens environmental impact, making the technology more sustainable for widespread use in various applications. However, a more detailed examination of cytotoxicity is warranted. Future research could focus on in vivo biocompatibility testing to further assess the safety and environmental impact of these composites.

## Conclusion

Hydroxyapatite is widely used as biomaterial and for biomedical applications. This work introduces a straightforward approach to utilizing hydroxyapatite in healthcare applications. The hydroxyapatite was decorated with different metal nanoparticles, Cu, Zn, and Ag at different loading ratios. The synthesized hydroxyapatite-metal base composites were tested against different bacterial and fungal strains to assess their microbial inhibition capabilities. The results demonstrated an effective method for decorating hydroxyapatite with Cu, Zn, and Ag. The loading ratios ranged from 0 to 15 wt% with homogeneous distribution on the surface of the hydroxyapatite. The prepared metal-hydroxyapatite composites showed a notable microbial inhibition on different strains of bacteria and fungi. The highest inhibition values were recorded for the 5% Ag/HAp formulation, showing values of 19.7 mm and 13.8 mm against *E. coli* and* S*.* aureus*, respectively. The 5% Ag/HAp concentration may strike an ideal balance, providing high antimicrobial activity without adverse effects on biocompatibility or material stability. These findings suggest that the HAp-based composites are highly recommended for infection prevention, particularly in healthcare settings where they could be applied to tools, linens, staff uniforms, and patient garments to reduce the spread of infections. Furthermore, as a future work, the developed HAp-based composites should be evaluated after doping with healthcare settings, and medical implants.

## Electronic supplementary material

Below is the link to the electronic supplementary material.

Supplementary Material 1

## Data Availability

The datasets used and/or analysed during the current study available from the corresponding author on reasonable request.

## References

[1] Borkow, G. & Gabbay, J. Biocidal textiles can help fight nosocomial infections. *Med. Hypoth.* **70**(5), 990–994. 10.1016/J.MEHY.2007.08.025 (2008). 10.1016/j.mehy.2007.08.025 17959322

[2] Collier, M. Silver dressings: More evidence is needed to support their widespread clinical use. *J. Wound Care* **18**(2), 77–78 (2009). 19418785

[3] Kangwansupamonkon, W., Lauruengtana, V., Surassmo, S. & Ruktanonchai, U. Antibacterial effect of apatite-coated titanium dioxide for textiles applications. *Nanomed. Nanotechnol. Biol. Med.* **5**(2), 240–249. 10.1016/j.nano.2008.09.004 (2009). 10.1016/j.nano.2008.09.004 19223243

[4] Ohko, Y. et al. Self-sterilizing and self-cleaning of silicone catheters coated with TiO2 photocatalyst thin films: A preclinical work. *J. Biomed. Mater. Res.* **58**(1), 97–101. 10.1002/1097-4636(2001)58:1%3c97::AID-JBM140%3e3.0.CO;2-8 (2001). 11153004 10.1002/1097-4636(2001)58:1<97::aid-jbm140>3.0.co;2-8

[5] Tarquinio, K. M. et al. Bactericidal effects of silver plus titanium dioxide-coated endotracheal tubes on pseudomonas aeruginosa and staphylococcus aureus. *Int. J. Nanomed.* **5**(April), 177–183 (2010). PMC2865012 20463933

[6] Liang, H. et al. Fabrication of tragacanthin gum-carboxymethyl chitosan bio-nanocomposite wound dressing with silver-titanium nanoparticles using freeze-drying method. *Mater. Chem. Phys.* **279**, 125770. 10.1016/j.matchemphys.2022.125770 (2022).

[7] Wang, F., Mu-Sen, L., Yu-Peng, L., Yong-Xin, Q. & Yu-Xian, L. Synthesis and microstructure of hydroxyapatite nanofibers synthesized at 37°C. *Mater. Chem. Phys.* **95**(1), 145–149. 10.1016/j.matchemphys.2005.05.034 (2006).

[8] Koutsopoulos, S. Synthesis and characterization of hydroxyapatite crystals: A review study on the analytical methods. *J. Biomed. Mater. Res.* **62**(4), 600–612. 10.1002/jbm.10280 (2002). 12221709 10.1002/jbm.10280

[9] Phan, B. T. N. et al. Synthesis and characterization of nano-hydroxyapatite in maltodextrin matrix. *Appl. Nanosci.* **7**(1), 1–7. 10.1007/s13204-016-0541-z (2017).

[10] Xiao, F., Ye, J., Wang, Y. & Rao, P. Deagglomeration of HA during the precipitation synthesis. *J. Mater. Sci.* **40**(20), 5439–5442. 10.1007/s10853-005-1919-6 (2005).

[11] Karimi, M. et al. Fabrication of shapeless scaffolds reinforced with baghdadite-magnetite nanoparticles using a 3D printer and freeze-drying technique. *J. Mater. Res. Technol.* **14**, 3070–3079. 10.1016/j.jmrt.2021.08.084 (2021).

[12] Currey, J. Sacrificial bonds heal bone. *Nature* **414**(6865), 699. 10.1038/414699a (2001). 11742376 10.1038/414699a

[13] Murugan, R. & Ramakrishna, S. Coupling of therapeutic molecules onto surface modified coralline hydroxyapatite. *Biomaterials* **25**(15), 3073–3080. 10.1016/j.biomaterials.2003.09.089 (2004). 14967541 10.1016/j.biomaterials.2003.09.089

[14] Gupta, R. & Xie, H. Nanoparticles in daily life: Applications, toxicity and regulations. *J. Environ. Pathol. Toxicol. Oncol. Off. Organ Int. Soc. Environ. Toxicol. Cancer* **37**(3), 209–230. 10.1615/JEnvironPatholToxicolOncol.2018026009 (2018). 10.1615/JEnvironPatholToxicolOncol.2018026009 PMC6192267 30317972

[15] Riaz, U., Shazia, I., Laila, S., Tayyaba, S. & Waleed, M. A. Chapter 8—health and safety hazards of nanomaterials. In *Micro and Nano Technologies*, edited by Muhammad Bilal Tahir, Muhammad Sagir, and Abdullah M B T - Nanomaterials: Synthesis Asiri Characterization, Hazards and Safety, 223–40. Elsevier. 10.1016/B978-0-12-823823-3.00012-4 (2021).

[16] Abdel-Aal, S. et al. Synthesis of agglomerated hydroxyapatite nanospheres with performance as anti-viral material. *Int. J. Mater. Technol. Innov.* **1**(1), 1–17. 10.21608/ijmti.2021.181066 (2021).

[17] Hamouda, H. I., Abdel-Ghafar, H. M. & Mahmoud, M. H. H. Multi-walled carbon nanotubes decorated with silver nanoparticles for antimicrobial applications. *J. Environ. Chem. Eng.*10.1016/j.jece.2021.105034 (2021).

[18] Abdel-Ghafar, H. M. & Hamouda, H. I. Development of an anti-organic fouling photothermal membrane for sustainable freshwater generation from wastewater. *Environ. Process.* **11**(2), 31. 10.1007/s40710-024-00709-3 (2024).

[19] Yuranova, T. et al. Antibacterial textiles prepared by RF-plasma and vacuum-UV mediated deposition of silver. *J. Photochem. Photobiol. A Chem.* **161**(1), 27–34. 10.1016/S1010-6030(03)00204-1 (2003).

[20] Das, R. & Amit, K. G. “Chapter 10 - Role and Importance of Hydroxyapatite in the Healthcare Sector.” In *Micro and Nano Technologies*, edited by Shadpour Mallakpour and Chaudhery Mustansar B T - Industrial Applications of Nanoceramics Hussain, 159–207. Elsevier. 10.1016/B978-0-323-88654-3.00011-1 (2024).

[21] Habib, M. L., Disha, S. A., Hossain, M. S., Uddin, M. N. & Ahmed, S. Enhancement of antimicrobial properties by metals doping in nano-crystalline hydroxyapatite for efficient biomedical applications. *Heliyon* **10**(1), e23845. 10.1016/j.heliyon.2023.e23845 (2024). 38192860 10.1016/j.heliyon.2023.e23845 PMC10772636

[22] Phatai, P. et al. Zinc-silver doped mesoporous hydroxyapatite synthesized via ultrasonic in combination with sol-gel method for increased antibacterial activity. *Sustainability* **14**(18), 11756 (2022).

[23] Nenen, A. et al. Synthesis of antibacterial silver and zinc doped nano-hydroxyapatite with potential in bone tissue engineering applications. *Ceram. Int.* **48**(23), 34750–34759. 10.1016/j.ceramint.2022.08.064 (2022).

[24] Vijayaraghavan, P. et al. Preparation and antibacterial application of hydroxyapatite doped Silver nanoparticles derived from chicken bone. *J. King Saud Univ. Sci.* **34**(2), 101749. 10.1016/j.jksus.2021.101749 (2022).

[25] Bauer, A. W., Kirby, W. M., Sherris, J. C. & Turck, M. Antibiotic susceptibility testing by a standardized single disk method. *Am. J. Clin. Pathol.* **45**(4), 493–496 (1966). 5325707

[26] Hudzicki. Kirby-Bauer Disk Diffusion Susceptibility Test Protocol. *American Society for Microbiology.* https://asm.org/protocols/kirby-bauer-disk-diffusion-susceptibility-test-pro (2009).

[27] Alizade, H. et al. Comparison of disc diffusion, broth microdilution and modified hodge test susceptibility testing of *Escherichia coli* isolates to beta-lactam antibiotics. *Med. Lab. J.* **10**(2), 19–24. 10.18869/acadpub.mlj.10.2.19 (2016).

[28] Gajic, I. et al. Antimicrobial susceptibility testing: A comprehensive review of currently used methods. *Antibiotics (Basel)* **11**(4), 427. 10.3390/antibiotics11040427 (2022). 35453179 10.3390/antibiotics11040427 PMC9024665

[29] Hamouda, H. I. et al. Novel nanoblades of graphene oxide decorated with zinc oxide nanocomposite as a powerful anti-microbial active weapon. *Synth. Met.* **296**, 117349. 10.1016/j.synthmet.2023.117349 (2023).

[30] Medeiros, M. A. N., de Oliveira, D. C. N., Rodrigues, D. D. P. & de Freitas, D. R. C. Prevalence and antimicrobial resistance of salmonella in chicken carcasses at retail in 15 Brazilian cities. *Revista Panamericana de Salud Publica = Pan Am. J. Public Health* **30**(6), 555–560. 10.1590/s1020-49892011001200010 (2011). 10.1590/s1020-49892011001200010 22358402

[31] Kim, Y. G., Seo, D. S. & Lee, J. K. Dissolution of synthetic and bovine bone-derived hydroxyapatites fabricated by hot-pressing. *Appl. Surface Sci.* **255**(2), 589–592. 10.1016/j.apsusc.2008.06.089 (2008).

[32] Heidari, F., Mohammad, E. B., Daryoosh, V. & Lobat, T. In situ preparation of iron oxide nanoparticles in natural hydroxyapatite/chitosan matrix for bone tissue engineering application. *Ceram. Int.* **41**(2, Part B), 3094–3100. 10.1016/j.ceramint.2014.10.153 (2015).

[33] Hu, W., Jun, M., Jianglin, W. & Shengmin, Z. Fine structure study on low concentration zinc substituted hydroxyapatite nanoparticles. *Mater. Sci. Eng. C* **32**(8), 2404–2410. 10.1016/j.msec.2012.07.014 (2012).

[34] Webster, T. J., Elizabeth, A.M.-S., Jennifer, L. S. & Elliot, B. S. Osteoblast response to hydroxyapatite doped with divalent and trivalent cations. *Biomaterials* **25**(11), 2111–2121. 10.1016/j.biomaterials.2003.09.001 (2004). 14741626 10.1016/j.biomaterials.2003.09.001

[35] Zykin, M. A. et al. Solid state solubility of copper oxides in hydroxyapatite. *J. Solid State Chem.* **262**, 38–43. 10.1016/j.jssc.2018.03.003 (2018).

[36] Heidari, F. et al. Mechanical properties of natural chitosan/hydroxyapatite/magnetite nanocomposites for tissue engineering applications. *Mater. Sci. Eng. C Mater. Biol. Appl.* **65**(August), 338–344. 10.1016/j.msec.2016.04.039 (2016). 27157760 10.1016/j.msec.2016.04.039

[37] Köhler, G. & Milstein, C. Continuous cultures of fused cells secreting antibody of predefined specificity. *Nature* **256**(5517), 495–497. 10.1038/256495a0 (1975). 1172191 10.1038/256495a0

[38] Kalpana, V. N. et al. Biosynthesis of zinc oxide nanoparticles using culture filtrates of aspergillus niger: Antimicrobial textiles and dye degradation studies. *OpenNano* **3**, 48–55. 10.1016/j.onano.2018.06.001 (2018).

[39] Shi, P. et al. Characterization of natural hydroxyapatite originated from fish bone and its biocompatibility with osteoblasts. *Mater. Sci. Eng. C Mater. Biol. Appl.* **90**(September), 706–712. 10.1016/j.msec.2018.04.026 (2018). 29853142 10.1016/j.msec.2018.04.026

[40] Rajendran, A., Rakesh, C. B., Duraipandy, N., Kiran, M. S. & Deepak, K. P. Synthesis, phase stability of hydroxyapatite-silver composite with antimicrobial activity and cytocompatability. *Ceram. Int.* **40**(7), 10831–10838. 10.1016/j.ceramint.2014.03.075 (2014).

[41] Guo, X. et al. Effect of calcining temperature on particle size of hydroxyapatite synthesized by solid-state reaction at room temperature. *Adv. Powder Technol.* **24**(6), 1034–1038. 10.1016/j.apt.2013.03.002 (2013).

[42] Noori, A. et al. Exploring the various effects of Cu doping in hydroxyapatite nanoparticle. *Sci. Rep.* **14**(1), 3421. 10.1038/s41598-024-53704-x (2024). 38341449 10.1038/s41598-024-53704-x PMC10858896

[43] Tourbin, M. et al. Agglomeration of stoichiometric hydroxyapatite: Impact on particle size distribution and purity in the precipitation and maturation steps. *Powder Technol.* **360**, 977–988. 10.1016/j.powtec.2019.10.050 (2020).

[44] Moulder, J. F. *Handbook of X-Ray Photoelectron Spectroscopy*. Edited by Jill Chastain. Minnesota: Perkin-Elmer Corporation (1992).

[45] Guo, J. et al. High efficiency and stability of Au–Cu/hydroxyapatite catalyst for the oxidation of carbon monoxide. *RSC Adv.* **7**(72), 45420–45431. 10.1039/C7RA08781K (2017).

[46] Amedlous, A. et al. Copper loaded hydroxyapatite nanoparticles as eco-friendly fenton-like catalyst to effectively remove organic dyes. *J. Environ. Chem. Eng.* **9**(4), 105501. 10.1016/j.jece.2021.105501 (2021).

[47] de Lima, C. O. et al. Zn-doped mesoporous hydroxyapatites and their antimicrobial properties. *Colloids Surfaces B Biointerfaces*10.1016/j.colsurfb.2020.111471 (2021). 33257159 10.1016/j.colsurfb.2020.111471

[48] Lamkhao, S. et al. Synthesis of hydroxyapatite with antibacterial properties using a microwave-assisted combustion method. *Sci. Rep.* **9**(1), 4015. 10.1038/s41598-019-40488-8 (2019). 30850662 10.1038/s41598-019-40488-8 PMC6408465

[49] Selim, M. S., Hamouda, H., Hao, Z., Shabana, S. & Chen, X. Design of γ-AlOOH, γ-MnOOH, and α-Mn2O3 nanorods as advanced antibacterial active agents. *Dalton Trans.* **49**(25), 8601–8613. 10.1039/D0DT01689F (2020). 32543624 10.1039/d0dt01689f

