# Supplementary material for: Enhanced antimicrobial efficacy of hydroxyapatite-based composites for healthcare applications
Source: Sci Rep. 2024 Nov 2;14:26426. doi: 10.1038/s41598-024-76088-4 (PMC11531545; doi:10.1038/s41598-024-76088-4)
Supplement: Supplementary file 1 — Supplementary Material 1 [file 41598_2024_76088_MOESM1_ESM.docx]

**Enhanced Antimicrobial Efficacy of Hydroxyapatite-based Composites for Healthcare Applications**

Maher Hassanain^1, 2^, Hamdy Maamoun Abdel-Ghafar^1^^[[1]](#footnote-0)^*, Hamed I. Hamouda^3, 4^, Fouad I. El-Hosiny^2^, Emad M. M. Ewais^1^

^1^ Central Metallurgical Research and Development Institute (CMRDI), PO Box 87 Helwan, 11421 Cairo, Egypt

^2^ Chemistry Department, Faculty of Science, Ain Shams University,  Abbassia, Cairo, 11566, Egypt

^3^ Dalian Engineering Research Center for Carbohydrate Agricultural Preparations, Liaoning Provincial Key Laboratory of Carbohydrates, Dalian Institute of Chemical Physics, Chinese Academy of Sciences, CAS, Dalian, 116023, China

^4^ Processes Development Department, Egyptian Petroleum Research Institute, Nasr City 11727, Cairo, Egypt

**Table S1:** Particle size distribution analysis of the Cu/HAp composites at different ratios.

| **D value (µm)** | **HAp** | **5% Cu** | **10% Cu** | **15% Cu** |
| --- | --- | --- | --- | --- |
| **D_10_ (µm)** | 1.54 | 1.75 | 1.98 | 3.29 |
| **D_25_ (µm)** | 2.44 | 3.08 | 3.88 | 6.58 |
| **D_50_ (µm)** | 4.55 | 7.16 | 9.92 | 14.32 |
| **D_75_ (µm)** | 9.14 | 15.16 | 20.04 | 25.98 |
| **D_90_ (µm)** | 15.86 | 23.15 | 31.13 | 39.67 |
| **D_98_ (µm)** | 26.46 | 33.09 | 45.95 | 58.25 |

**Table S2:** Particle size distribution analysis of the Zn/HAp composites at different ratios.

| **D value (µm)** | **HAp** | **5% Zn** | **10% Zn** | **15% Zn** |
| --- | --- | --- | --- | --- |
| **D_10_ (µm)** | 1.54 | 1.75 | 2.18 | 2.01 |
| **D_25_ (µm)** | 2.44 | 3.01 | 4.20 | 3.94 |
| **D_50_ (µm)** | 4.55 | 6.62 | 9.30 | 9.70 |
| **D_75_ (µm)** | 9.14 | 1374 | 16.92 | 17.81 |
| **D_90_ (µm)** | 15.86 | 20.69 | 24.55 | 25.56 |
| **D_98_ (µm)** | 26.46 | 29.34 | 33.80 | 34.86 |

**Table S3:** Particle size distribution analysis of the Ag/HAp composites at different ratios.

| **D value (µm)** | **HAp** | **5% Ag** | **10% Ag** | **15% Ag** |
| --- | --- | --- | --- | --- |
| **D_10_ (µm)** | 1.54 | 1.86 | 1.87 | 1.84 |
| **D_25_ (µm)** | 2.44 | 3.35 | 3.39 | 3.36 |
| **D_50_ (µm)** | 4.55 | 7.79 | 8.12 | 8.41 |
| **D_75_ (µm)** | 9.14 | 15.67 | 16.25 | 17.65 |
| **D_90_ (µm)** | 15.86 | 23.53 | 24.31 | 27.45 |
| **D_98_ (µm)** | 26.46 | 33.35 | 33.85 | 40.52 |


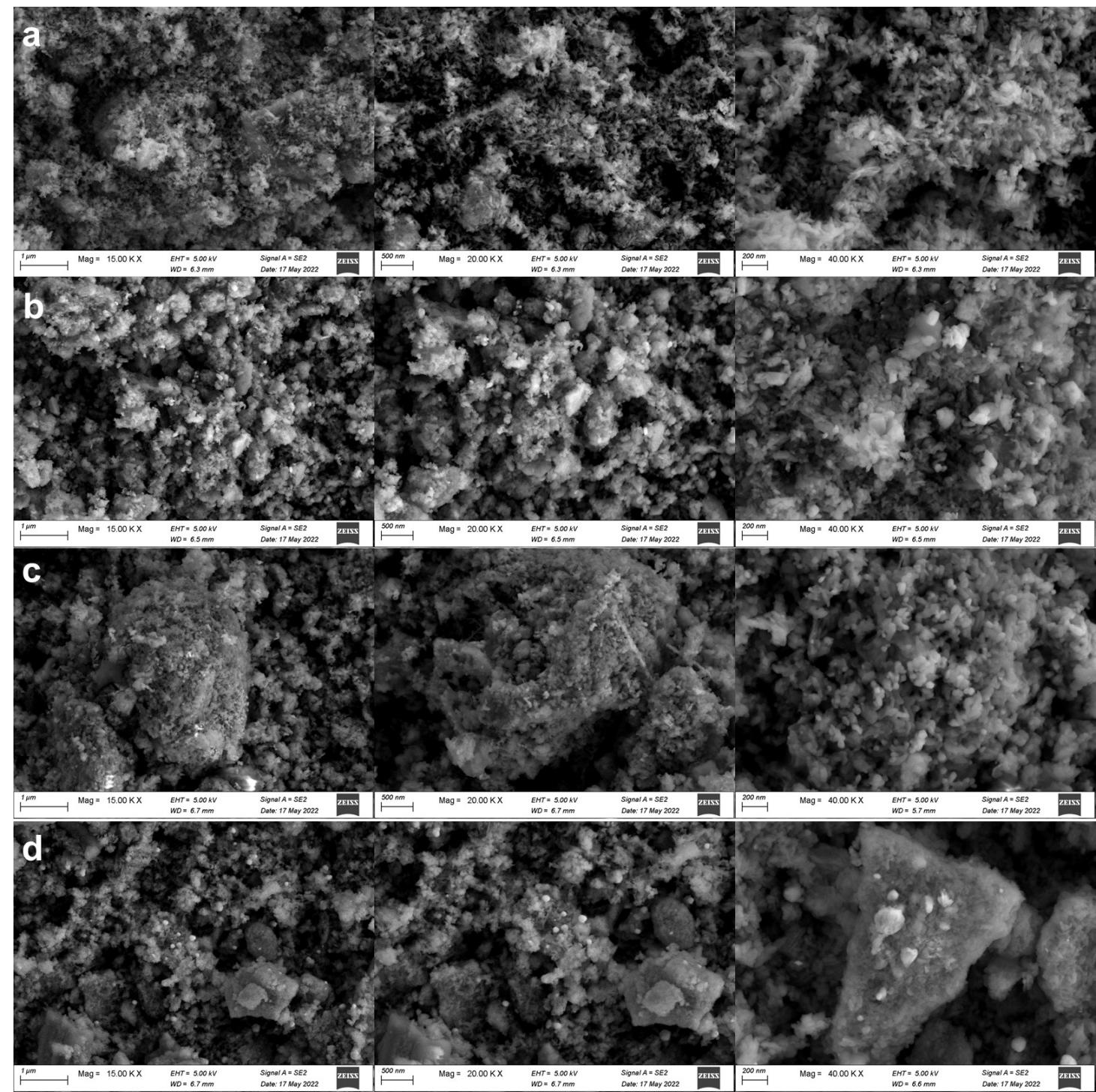


**Fig. S1.** SEM analysis of the (a) pristine HAp (b) 10% Cu/HAp, (c) 10% Zn/HAp, and (d) 10% Ag/HAp composites at different magnifications.


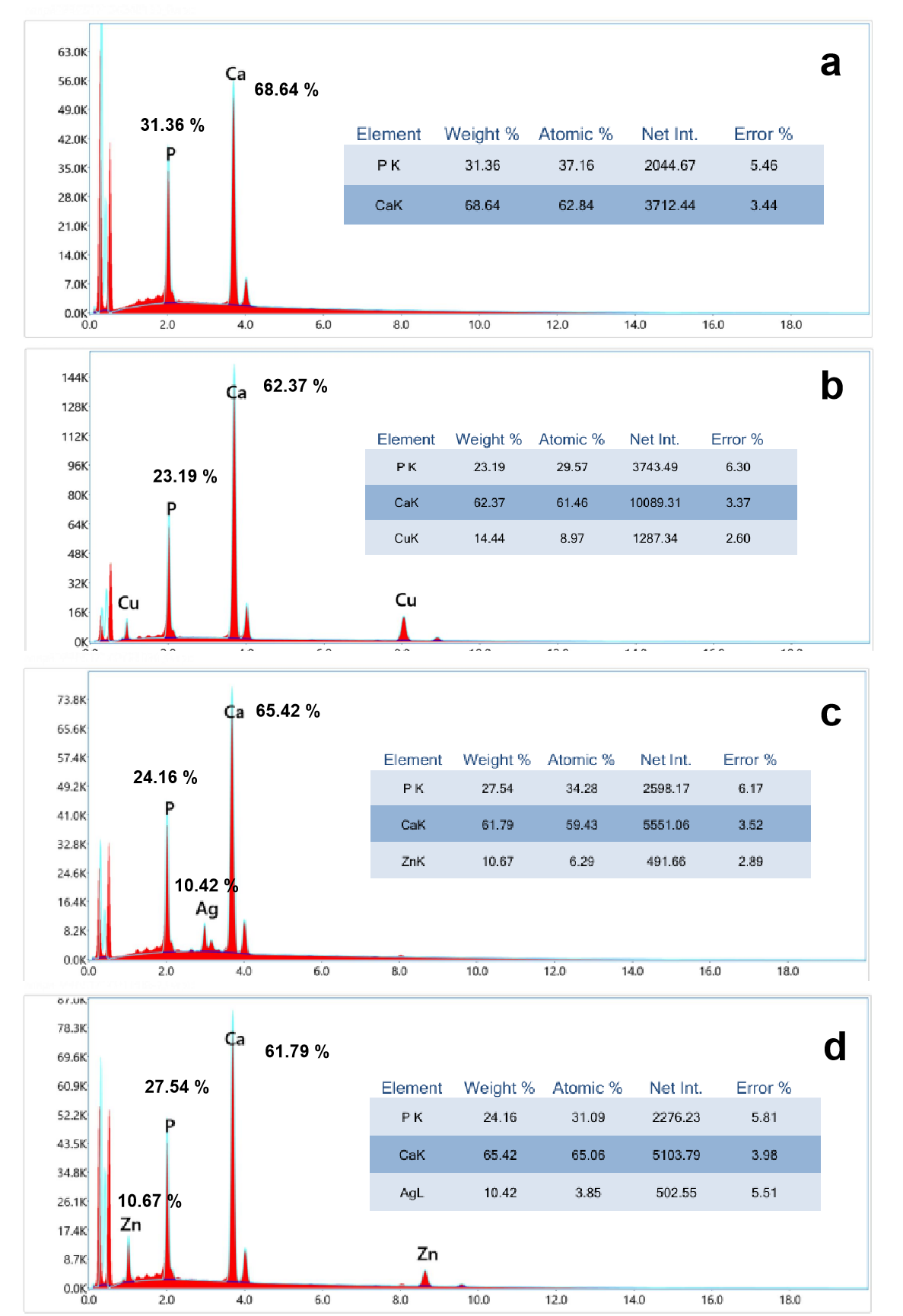


**Fig. S2** EDX analysis of the (a) pristine HAp, (b) 10% Cu/HAp, (c) 10% Zn/HAp, and (d) 10% Ag/HAp composites.

**
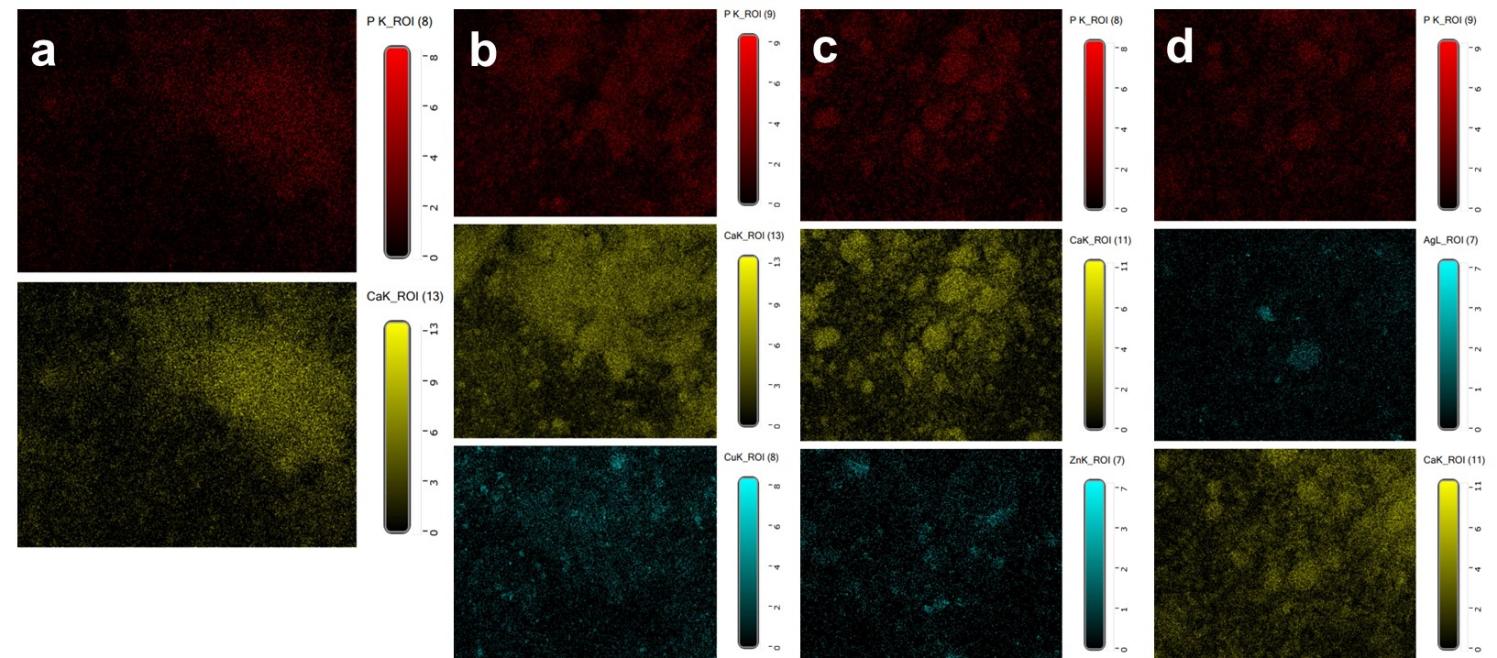
**

**Fig. S3.** SEM mapping of the elemental distribution of the (a) pristine HAp (b) 10% Cu/HAp, (c) 10% Zn/HAp, and (d) 10% Ag/HAp composites.

1. * **The corresponding author:** Hamdy Maamoun Abdel-Ghafar, E-mail addresses: [hamdy.maamoun@gmail.com](mailto:hamdy.maamoun@gmail.com) [↑](#footnote-ref-0)
